# Salt Fluxes in a Complex River Mouth System of Portugal

**DOI:** 10.1371/journal.pone.0047349

**Published:** 2012-10-10

**Authors:** Nuno Vaz, João D. Lencart e Silva, João Miguel Dias

**Affiliations:** CESAM, Departamento de Física, Universidade de Aveiro, Campus de Santiago, Aveiro, Portugal; Humboldt University, Germany

## Abstract

Measurements of velocity and salinity near the mouth and head of the Espinheiro channel (Ria de Aveiro lagoon, Portugal) are used to study the local variation of physical water properties and to assess the balance, under steady conditions, between the seaward salt transport induced by river discharge and the landward dispersion induced by various mixing mechanisms. This assessment is made using data sampled during complete tidal cycles. Under the assumption that the estuarine tidal channel is laterally homogeneous and during moderate tidal periods (except for one survey), currents and salinity data were decomposed into various spatial and temporal means and their deviations. Near the channel's mouth, the main contributions to the salt transport are the terms due to freshwater discharge and the tidal correlation. Near the channel's head, this last term is less important than the density driven circulation, which is enhanced by the increase in freshwater discharge. The remaining terms, which are dependent on the deviations from the mean depth have a smaller role in the results of salt transport. The computed salt transport per unit width of a section perpendicular to the mean flow is in close agreement to the sum of the advective and dispersive terms (within or very close to 12%). An imbalance of the salt budget across the sections is observed for all the surveys. Considerations are made on how this approach can inform the management of hazardous contamination and how to use these results to best time the release of environmental flows during dry months.

## Introduction

Tidal estuaries and channels are often located close to heavily populated areas where waste water disposal from urban and industrial sources can degrade ecosystem health affecting the services it provides and its resilience to further impacts. The transport mechanisms of contaminated water in these systems are closely linked to the transport of a natural tracer: salt. The diffusion and advection of freshwater out of the estuary and the upstream flux of saltier ocean water are governed by the same mechanisms as any other soluble or suspended pollutant.

In a tidal channel, the main mechanisms driving the circulation and providing the turbulent energy for the mixing processes are the freshwater discharge and the barotropic and baroclinic components of the gradient pressure force due to tidal forcing and to the typical estuarine longitudinal salinity gradients, respectively [1]. In steady-state conditions, there is a balance between seaward advection of river runoff and landward mixing of saltier ocean water. Among the processes that cause mixing, longitudinal turbulent diffusion plays a minor role and the overall landward mixing is better termed dispersion rather than diffusion [1], [2] and [3]. The advective seaward salt transport is driven by the river flow momentum and volume input and by the horizontal density gradient. The landward salt transport is a consequence of the dispersion produced by the effects of tidal and wind mixing and by the same horizontal density gradient.

In estuarine regions there is some degree of heterogeneity of the main controls on the transport and flushing of pollutants. Understanding what controls salt transport in different zones of these systems helps to anticipate how natural and anthropogenic changes to the ecosystem affect its capacity to flush pollutants. Thus, changes to one control such as the amount of river runoff will have great impact in one region of the ecosystem (e.g. near the channel head) whilst in other parts of the system such change will have a minimal effect in transport (e.g. near the channel mouth). Studying the mechanisms controlling salinity is important since salinity is one of the most severe environmental factors affecting species distribution in estuaries and surrounding low-lying lands. Despite some views that salinity is natural and hence not a contaminant, it is now well recognized in the scientific literature that anthropogenic changes of salinity distribution can have profound and measurable effects on riverine ecosystems. In addition, understanding the salt transport mechanisms in tidal channels like the Espinheiro, can help identify risks of groundwater salt intrusion in surrounding agricultural fields.

**Figure 1:**
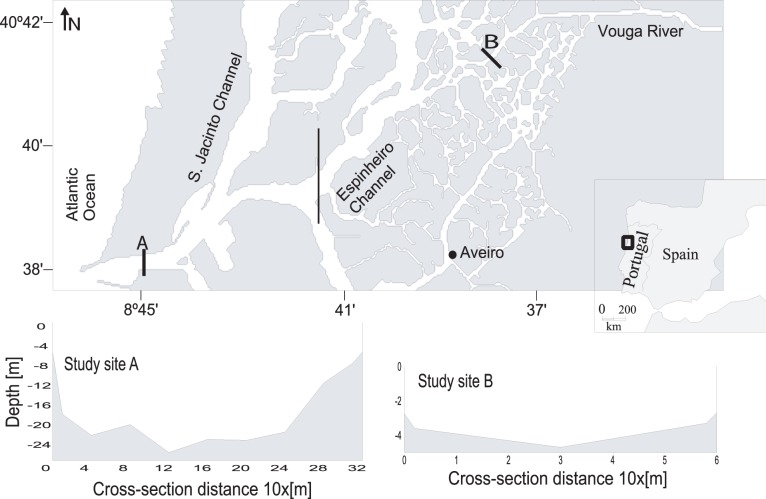
The Espinheiro channel showing the survey locations A and B.

The Espinheiro channel is one of the four main branches of the Ria de Aveiro, a mesotidal and shallow (mean depth of about 1 m relative to the local datum) coastal lagoon located in the northwest coast of the Iberian Peninsula (see Figure 1). This channel is located in the very complex central area of the lagoon and it includes two distinct regions: the first extending from the mouth of the lagoon to near the black solid line marked in Figure 1 and the second is the Espinheiro channel itself, extending from the referred line to near station B. About 2 km upstream, a small dam is closed during the dry summer months to protect the low-land of Baixo-Vouga (agricultural fields) and a paper mill waste-water treatment plant from salt intrusion, opening only for a short time interval during each ebb. For simplicity, the study area from the mouth of the lagoon (near station A) to the aforementioned dam upstream station B will be hereinafter referred as the Espinheiro channel. The study area is approximately 11 km long, has an averaged width of about 200 m and a mean depth, along its longitudinal axis, of about 10 m. The tides are mixed semi-diurnal (M_2_ represents about 90% of the tidal energy [4]).

Previous studies ([5], [6] and [7]) revealed that the tide is strongly distorted as it progresses upstream from the mouth toward the end of the channel, due to changes in channel's geometry and bathymetry. The general characteristics of the tidal wave are those of a damped progressive wave. Nevertheless, in shallow areas the tidal wave assumes the main characteristics of a standing wave.

From a dynamical point of view, the Espinheiro channel may be considered one of the most important areas of the Ria de Aveiro, due to the strongest currents observed there, reaching values higher than 2 ms^−1^ [8]. Conversely, the lagoon's remaining channels are very shallow, leading to a strong dumping of the current.

The estimated tidal prism for the lagoon at extreme spring and extreme neap is according to [9] 136.7×10^6^ and 34.9×10^6^ m^3^, respectively. Close to the black solid line this volume is about 40×10^6^ at extreme spring and 15×10^6^ m^3^ at extreme neap tide [9]. The total estimated freshwater input for the lagoon is very small (about 1.8×10^6^ m^3^ during a complete tidal cycle) [10] when compared to the tidal prism, both at the mouth and at the beginning of Espinheiro channel.

The largest freshwater contribution is from the Vouga River. Its mouth is located near the head of the study area and therefore its freshwater inflow is determinant in the establishment of the channel's physical patterns. There is a study by [11] presenting flood fresh water values of 3400 m^3^s^−1^ for a return period of 25 years. The average flow for Vouga River is referred by [12] as 25 m^3^s^−1^, but according to [11] this value is underestimated. From September 2003 to September 2004, measurements of the Vouga river's incoming flow were performed by the authors under the scope of this work and the annual averaged flow value found was 31.45 m^3^s^−1^ [13]. The highest river flow values occurred during autumn and the beginning of the winter with a maximum of 143.16 m^3^s^−1^ in January. The minimum flows occurred during the warmer seasons, with values ranging from 30 m^3^s^−1^ (early Spring) to 2.0 m^3^s^−1^ (late summer).

In this paper, the effect of the salinity gradient caused by freshwater discharge and tidal stirring is investigated in the transport of dissolved substances. The main objective of this work is to establish the relative importance of the several terms of the equation of salt transport in each of the two study areas. This will be carried out by studying the local variations of physical water properties and estuarine circulation, and by estimating the advective and dispersive salt transport through a unit width across the section passing through the fixed stations A and B, located near the mouth of the lagoon and 8 km upstream, respectively (see Figure 1).

## Materials and Methods

### Current and Salinity Measurements

As a preamble we should declare that no specific permits were required for the described field studies. Moreover all the data were surveyed at non-privately-owned locations and this study did not involve endangered or protected species.

Throughout this paper, salinity values are expressed in “Pratical Salinity Units” (psu), which is defined as conductivity ratio with no units. A seawater sample with a conductivity ratio of 1.0 at 15°C with a KCl solution containing a mass of 32.4356 g in a total mass of 1 kg of solution has a salinity of 35 (UNESCO, 1985) [14].

Vertical profiles of salinity and current velocity were sampled near the mouth of the channel (station A –40°38.66'N, 8°44.91'W) and near the channel's head (station B –40°41.29'N, 8°39.03'W) (see Figure 1). In both locations the measurements were made at an anchor station placed in the middle of the channel. In these two locations, the cross section has a regular geometry and the salinity differences between the two channel's margins are always less than 1. Therefore, it seems to be fair the assumption of a laterally homogeneous channel. At station A, two surveys were performed during a neap and the following spring tide (February 2002). At station B, 4 surveys were performed, one for each season of the year, during neap tide. The measurements were performed at lunar hourly intervals, for a total of 13 (station A surveys) and 25 sampling times (station B surveys).The depths of stations A and B (during high tide) are 25 and 6.5 m, respectively. The main tidal and river flow characteristics for the sampled periods are summarized in Table 1.

**Table 1:** Summary of tidal and river discharge conditions during the survey periods.

Date	River flow [m^3^s^−1^]	Tide	Tidal range [m]
21/02/2002	–	Neap	1.0
28/02/2002	–	Spring	3.0
15/01/2004	58.0	Neap	1.7
16/06/2004	11.2	Neap	2.0
27/08/2004	3.0	Neap	1.8
03/1272004	5.0	Neap	1.6

The measurements at station A were taken during one tidal cycle in each survey and were performed by the Hydrographic Institute of the Portuguese Navy. For the current velocity measurements it was used an Acoustic Doppler Currentmeter Profiler (ADCP) Workhorse Sentinel (RD Instruments) with a frequency of 614.4 KHz. This instrument measures current velocity in a range from 0 to 5 ms^−1^ with a resolution and an accuracy of 0.001 ms^−1^ and 0.25% of the water velocity relative to the ADCP, respectively. A CTD Idronaut model 316 was used to measure salinity. This instrument measures water temperature in a range between −3 and 50°C with a resolution and an accuracy of 0.005 and 0.003°C, respectively. The conductivity is measured between 0 and 64 mS/cm with a resolution and accuracy of 0.001 and 0.003 mS/cm. The salinity is calculated from water temperature and conductivity data using the Practical Salinity Scale 1978 (PSS78).

The measurements at station B were taken during two consecutive tidal cycles, using a SAIV A/S mini STD model SD204 to measure salinity and a Valeport current meter model 105 to measure velocity. The STD measures water temperature using a thermistor which works in a range from −2 to 40°C with an accuracy of +/−0.01°C. The water pressure is measured using a piezoresistive sensor with an accuracy of +/−0.02% of the maximum operation depth (500 m) and for the conductivity the instrument uses an inductive cell working in a range between 0 and 70 mS/cm with an accuracy of +/−0.02 mS/cm. The salinity is calculated from the conductivity, water temperature and water pressure in a range from 0 to 40 with an accuracy of 0.02. The current meter has a high impact styrene impeller to measure the current speed in a range of 0.1 to 5 ms^−1^ with an accuracy of ±2.5% of reading above 0.5 ms^−1^ and ±0.01 ms^−1^ below 0.5 ms^−1^. The current direction is measured using a flux gate compass with a range from 0–360° with a resolution of 0.5°.

River flow values concurrent with station B surveys were estimated from current speed obtained several km upstream from station B, 3 hours after the low tide hour predicted for the lagoon's mouth. This procedure guarantees that the measurements of current speed were made outside the region of tidal flood influence. The current velocity data were collected using the Valeport current meter model 105 previously referred. In order to compute the flow, the river section was divided in twenty four segments of 2 m and the current velocity and the water depth were measured for each segment. The total river flow was obtained adding all individual segment results.

The current velocity was calculated along the main flow direction from original data of current velocity intensity and direction. The main direction velocity component (*V* component) and the salinity profiles were combined into a simultaneous lunar hourly measurements data set with constant depth intervals of 0.5 (station A) and 0.2 m (station B), respectively. The sampling depth of each measurement was reduced to a non-dimensional depth [*Z  =  z/h*(*t*)] of 1/10, taking into account the local water depth [*h*(*t*)], to minimize the sampling water depth variations during the tidal cycle [15]. Down the water column, the measurements were interpolated from surface to bottom, in depth intervals of *Z*/10. The surface and the bottom values were weighted one-half of the others when averaging over depth. At station B, 2 consecutive tidal cycles of 12.421 h (the *M_2_* period) were sampled every lunar hour (1.04 solar hours), and the tidal average taken by giving the weight of 1 to every measurement except for the 1^st^ and last which were given a weight of 0.5.

The possible errors in the computation of the salt transport were obtained using the methodology followed by [16] and [17] by re-running the previous procedures using the bands of sensor accuracy of the used equipment.

### Salt Transport and Vertical Stability

#### Salt transport theory

The formulation used for the instantaneous advective mass transport of salt per unit width of a section, normal to the longitudinal flow of the estuarine channel, is equal to
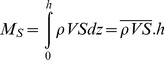
(1)where *ρ* is the density, *V* and *S* are the longitudinal velocity component and salinity, respectively. The upper bar denotes averaging over the total depth of the water column, *h*. In the International System of Units (SI), the salt transport comes in kgm^−1^s^−1^.

The non-tidal salt transport (*T_S_*) over one or more tidal cycles (*T*) is given by
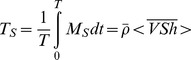
(2)where the mean density 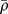 is assumed to be constant and the angle brackets denote averaging over one or more tidal cycles. The time interval *T* is a multiple integer of the tidal period.

In the case of a tidal flow, only a part of the transport is described by the product of the tidal and depth averages of the longitudinal component of the velocity (*V* velocity) and salinity. Turbulent fluctuations, which have time scales smaller than 1 minute, were not measured since only time scales of minutes, or longer, are of interest for estuaries [3]. The correlations between fluctuating salinity and velocity at a given point may be obtained through the decomposition of the instantaneous salinity and velocity profiles into their mean, tidal, steady and deviation terms. For a laterally homogeneous estuary or when the salt transport is calculated per unit width of a section perpendicular to the mean flow at time *t*, these profiles may be written as,
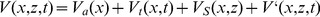
(3)

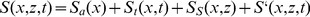
(4)where the mean component 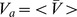, the tidal (barotropic) component 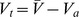, the steady (steady-state depth-varying baroclinic vertical velocity) component 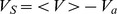, and the deviation component 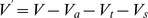. Similar expressions are valid in the *S_a_, S_t_, S_s_ and S’* computation.

The local depth *h(x,t)* at the anchor station varies with the tidal height and may be decomposed into two components [1]:
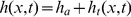
(5)where *h_a_*  =  <*h*> is the time-average water depth and *h_t_(x,t)* is the tidal height.

Introducing equations (3), (4) and (5) into equation (2), the advective salt transport under steady-state conditions may be decomposed into 32 terms [1]. Many of these terms vanish or may be neglected upon taking their depth and time averages ([1], [2]; [3]; [18] and [19]) since, by definition, 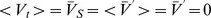, and similarly for salinity. Only the product of similar *V* and *S* components is considered. Other terms which have no physical expression, since no expected correlations between steady, tidal and deviation components exists, are neglected. This leaves seven terms, and therefore the total salt transport, per unit width, during a tidal cycle is described by,
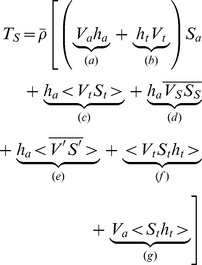
(6)


The first two terms of equation (6) represent the seaward salt advection by the mean current and Stokes wave transport. These two terms ((a) and (b)), taken together account for the transport of mean salinity by freshwater discharge. This process flushes the estuary clear of salt and sharpens the frontal gradient between the river and ocean [3]. The remaining terms are considered to represent landward dispersion of salt through mixing by various processes, decreasing the frontal gradient. Term (d), the correlation between tidal salinity and current, transports freshwater downstream and salt upstream which in mixed and partially mixed estuaries does so through dispersion. When acting in the same direction of (a) and (b) this term increases frontal gradient, resulting into convective overturning conversely [3]. Several authors related these terms to certain physical mechanisms which are listed in Table 2 [1]; [2]; [3]; [18], [19] and [20].

**Table 2:** Physical processes related with the terms of equation 6.

Term	Physical processes
(a)	Freshwater discharge or residual velocity
(b)	Stokes drift or progressive tidal wave transport
(c)	Topographic trapping
(d)	Gravitational circulation, bathymetric tidal pumping, steady wind effect
(e)	Tidal shear and unsteady wind effect
(f)	Tide dispersion via triple correlation
(g)	Net advection of cross correlation between salt and tide

Under steady conditions, there is no net transport and the sum of the right hand side terms of equation (6) should be zero. Equations (2) and (6) are distinct mathematical expressions of the same quantity. Therefore, in order to check the computational procedure and if the neglected terms are in fact small, a comparison of the net salt transport results computed using both equations will be used.

#### Water column stability

Here, the water column stability during a tidal cycle is investigated through the determination of the Richardson number. In conditions such as ours, where the precise determination of the density gradients is impractical, the layer Richardson number can be used instead:
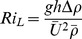
(7)where, *h = h(t)* is the local depth, Δ*ρ* is the difference between the seabed and surface density and 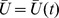 is the depth averaged velocity. [21] and [22] defined *Ri_L_* = 20 as the upper limit for the occurrence of turbulent mixing near the halocline in partially mixed estuaries.

For *Ri_L_*>20 mixing is negligible. When this value is smaller than 20 the bottom turbulence increases, and the stratification decreases. If *Ri_L_*<2 the turbulence becomes isotropic and mixing is fully developed [18]. Using this range (2<*Ri_L_*<20) as a simplified mixing criterion, it is possible to evaluate the spatial and temporal variation of the vertical stability of the water column. This method was used by [23] and [24] to study the gravitational stability at anchor stations.

## Results and Discussion

### Currents and Salinity

**Figure 2:**
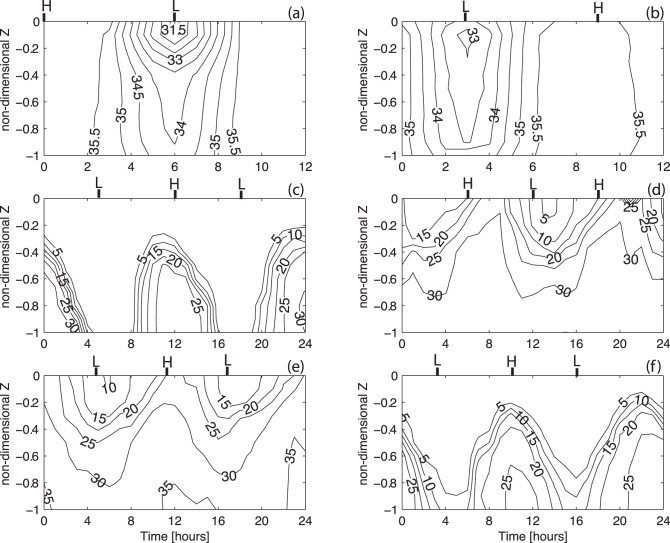
Contour figures of salinity as a function of the non-dimensional depth (*Z*) and time for all survey periods. *H* and *L* indicate the local high and low-water. (a) 21/02/2002 (station A); (b) 28/02/2002 (station A); (c) 15/01/2004 (station B); (d) 16/06/2004 (station B); (e) 27/08/2004 (station B); (f) 03/12/2004 (station B).

**Figure 3:**
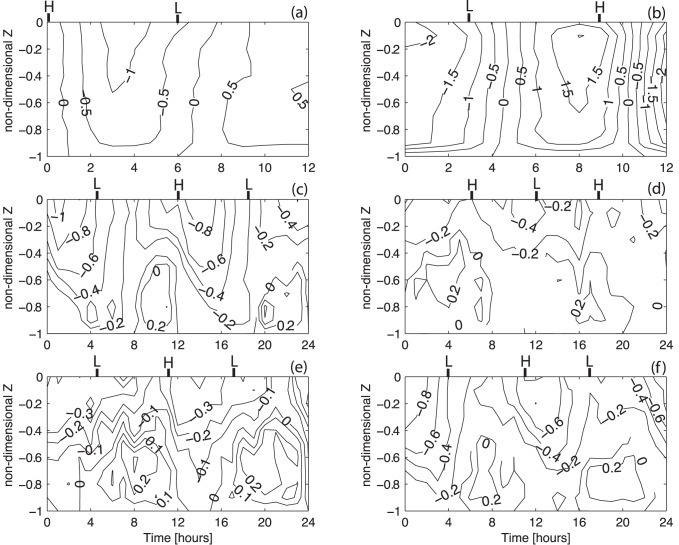
Contour figures of longitudinal velocity component as a function of the depth (Z) and time for all survey periods. *H* and *L* indicate the local high and low-water. (a) 21/02/2002 (station A); (b) 28/02/2002 (station A); (c) 15/01/2004 (station B); (d) 16/06/2004 (station B); (e) 27/08/2004 (station B); (f) 03/12/2004 (station B).

Figures 2 and 3 show the temporal evolution of the salinity and longitudinal velocity profiles for each of the surveys. Figures 2a and 2b show no vertical stratification. The lower salinity values appear near low-water. During the February 21^st^ survey, the water column is always well mixed except during the two hours before and after low-water when the difference between surface and bottom salinity is 2.5. Near the mouth of the channel, the velocity maximum is reached during the ebb, with values of −1.0 and −2.0 ms^−1^ as shown in Figures 3a and 3b.

**Figure 4:**
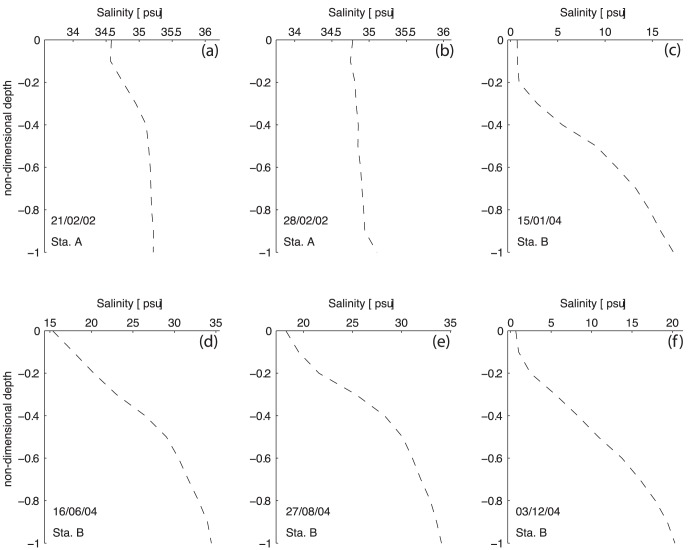
Profiles of non-tidal salinity at stations A and B.

**Figure 5:**
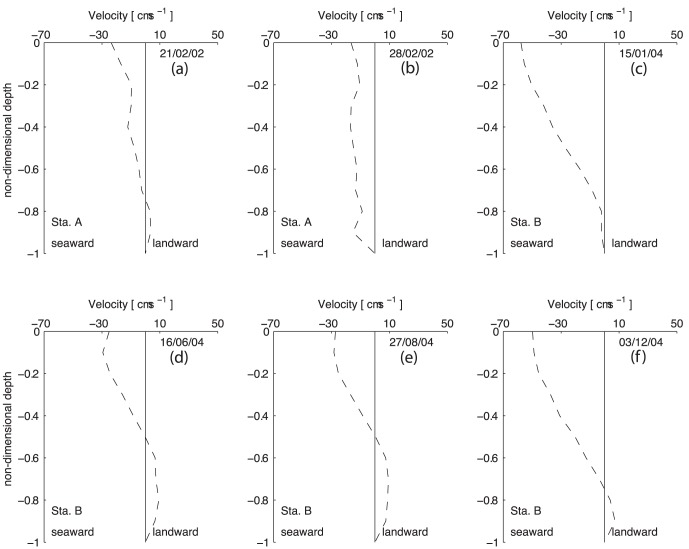
Profiles of non-tidal velocity at stations A and B.

The profiles of tidally-averaged velocity and salinity are shown in Figures 4 and 5. Station A results reveal almost no density-driven circulation, especially during the second survey (February 28^th^). The difference between surface and bottom salinity is lower than 1 (in both surveys) (Figures a and b). The tidally-averaged velocity is seaward through most of the water column. Near the surface, the velocity maximum is −0.24 and −0.17 ms^−1^ during the first and second surveys, respectively. During the first survey, an inversion of the tidally-averaged velocity is visible close to the bottom (Z = −0.7). The layer Richardson number presents values lower than 2 and increasing to 20 during within first hour before and after low-water, revealing a well-mixed water column. For the February 28^th^ survey, the layer Richardson number presents values lower than 20 during all the tidal cycle, meaning that no stratification is observed.

Station B station was placed closed to the mouth of the Vouga River, and therefore the circulation is strongly influenced not only by the tidal flow but also by the freshwater inflow. Furthermore, this location is narrow (approximately 60 m wide) and relatively shallow (maximum of 6.5 m depth during high-water in this location). The tidal and freshwater inflows are summarized in Table 1. The spring and summer results are influenced by the existence of a small dam 2 km upstream of station B.

During the January and December surveys, the estimated freshwater inflow was 58 and 5 m^3^s^−1^, respectively. The measured changes in salinity and velocity (Figures 2c, 2f, 3c and 3f) show that events leading to either vertical stratification or mixing occur within the period sampled. The stratification increases during the flood, reaching its maximum one hour before the high-water. Stratification decreases during the ebb until the local low-water. The entire water column is filled with freshwater spreading from the river during the beginning of the flood tide. During the December survey, when the discharge from the river is lower, the water column shows salinity values of 5 during the beginning of the flood. The vertical lunar hourly profiles of the longitudinal velocity indicate an asymmetry between the flood (∼V>0) and the ebb (∼V<0) currents. The ebb currents are stronger than the flood currents, reaching maxima of −1.0 and 0.3 ms^−1^ during the January survey and −0.8 and 0.3 ms^−1^ during the December survey, respectively. A similar velocity asymmetry was observed by [25] in studying the dynamics of a tropical estuary, the Curimataú River, NE Brazil. The tidally-averaged velocity profiles show that the current is seaward throughout the water column (Figure 5c) with the exception of an inversion near the bottom (Z = −0.8) during the December survey (Figure 5f). The surface maxima are −0.58 and −0.5 ms^−1^ during January and December, respectively. The tidally-averaged salinity profiles show values close to 0 near the surface increasing in depth to values of 17.2 (January) and 20.3 (December).

**Figure 6:**
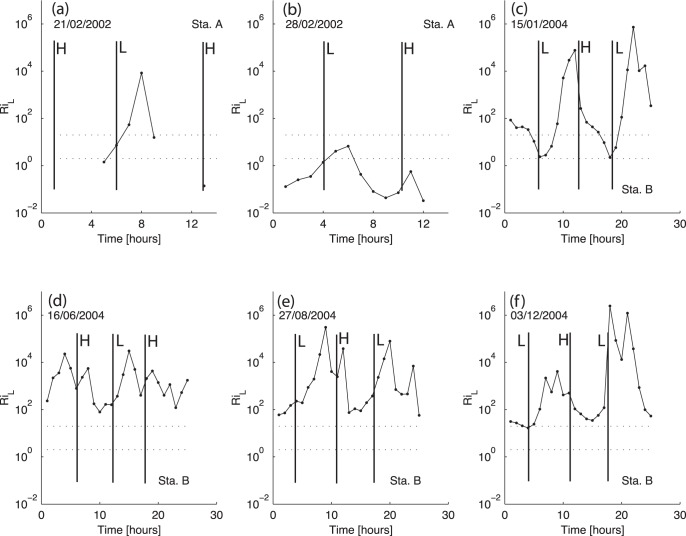
Temporal variation of the layer Richardson number. The dotted lines (*Ri_L_* equal to 2 and 20) are the limits of weak vertical stability.

The layer Richardson number shows a reduction in stratification (with values lower than 20) in January (Figure 6c) at low-water. The maximum values are reached one hour before high-water when the stratification is fully developed. For December (Figure 6f) this number is higher than 20, revealing the high stability of the water column, except around low-water, when turbulent mixing leads to a reduction in stratification.

During the spring and summer campaigns, the salinity data shows a stratified water column during all the sampling period. Although, the estimated freshwater inflow of 11.2 and 3.0 m^3^s^−1^ during the June and August campaigns is higher than in December, during summer the water column is saltier than in winter. This feature is due to the effect of the dam located 2 km upstream which is open only during a short period of time during each ebb. In fact, the freshwater is stored upstream of the dam and released only during a limited time during the ebb tide, as indicated by the salinity minimum at low-water of 5 and 10 (Figure 2d and 2e, respectively). Close to the channel's bed, the salinity is always higher than 30 revealing the presence of oceanic water. The velocity record (Figures 3d and 3e) shows low velocity values, ranging from −0.4 and 0.2 ms^−1^ during June and −0.3 and 0.2 ms^−1^ during the August campaign. Once more, the asymmetry between ebb and flood currents is observed. The vertical profiles of tidally-averaged velocity and salinity confirm the aforementioned results. Currents are stronger during the ebb with maxima surface values of −0.29 (Figure 5d) and −0.27 ms^−1^ (Figure 5e). An inversion in the velocity profile is observed near the middle of the water column (Z = −0.6) (ebb to flood currents) with maxima of 0.093 (Figure 5d) and 0.072 ms^−1^ (Figure 5e) for the June and August surveys, respectively. The salinity profiles show brackish surface values with maxima of 15.3 and 18.2 during the June and August surveys, respectively. These values increase toward the bottom until values higher than 34. During these two campaigns, the layer Richardson number is always higher than 20, revealing a highly stable water column (see Figure 6d and 6e).

### Salt Transport Results

The salt transport method presented in section “Salt transport and vertical stability” has been applied to compute the relative contribution of the individual terms of equation 6 in systems with several degrees of stratification. The net salt transport and its advective and dispersive components obtained from the application of this methodology for stations A and B are show in Table 3. As a check on the use of equation 6, to evaluate accuracy of the computational method, a calculation was made using equation 2 and the results compared. The two methodologies agree within 12%, which is an acceptable difference according to [1] and [3]. The exception are the January results, when a high river inflow (that fills the water column with freshwater) combined with a spring tide (which advects saline water upstream) gives to Station B location some salt wedge characteristics. The agreement between both calculations indicates that the terms omitted to obtain equation 6 were small.

**Table 3:** Salt transport in the Espinheiro channel [kgm^−1^s^−1^].

Location	Date	(a)	(b)	(c)	(d)	(e)	(f)	(g)	∑ (a)–(g)	Equation 2
A	21/02/2002	−59.33±0.51	2.95±0.10	5.53±0.31	0.35±0.01	0.34±0.01	0.00	−0.02±0.001	−50.18±1.00	−50.17±1.00
A	28/02/2002	−105.48±0.61	23.87±0.80	14.42±0.82	0.04±0.01	0.44±0.01	−0.06±0.001	−0.09±0.005	−66.86±1.21	−66.88±1.21
B	15/01/2004	−12.61±0.41	0.43±0.01	1.81±0.04	6.78±0.14	3.97±0.05	−0.033±0.001	−0.98±0.03	−0.67±0.20	−0.16±0.16
B	16/06/2004	−8.08±0.52	0.46±0.01	−0.03±0.02	4.00±0.20	0.54±0.03	0.00	−0.11±0.01	−3.22±0.33	−3.51±0.34
B	27/08/2004	−7.96±0.57	0.94±0.06	−0.12±0.01	3.48±0.17	0.22±0.02	0.00	−0.12±0.01	−3.56±0.38	−3.71±0.38
B	03/12/2004	−11.26±0.39	0.33±0.01	0.27±0.01	6.85±0.18	1.51±0.02	−0.030±0.002	−0.49±0.02	−2.82±0.20	−2.58±0.20

Letters (a) to (g) refer to Equation 6 salt transport terms.

According to the results, the main contribution to the salt transport is from term (a). The sum of term (a) and (b) over a tidal cycle, which represents top to bottom bulk advection of salt, is negative for all surveys indicating a seaward (advective) salt transport due to the tidally-averaged velocity induced by the river discharge. At station A, term (a) almost doubles from the neap tide of February 21^st^ to the following spring tide (−59.33±0.51 to −105.48±0.61 kgm^−1^s^−1^). At station B, this term is higher during the January and December surveys than during the June and August surveys, presenting a maximum of −12.61±0.41 during the January survey and a minimum of −7.96±0.57 kgm^−1^s^−1^ during the August survey.

Term (b) is positive for all the surveys. Having a positive sign represents a landward (dispersive) tidal wave transport of salt. In this channel, as in many others estuaries and tidal channels, there is a progressive wave component, which means that the high-water occurs at maximum flood velocity and low-water at maximum ebb velocity. So, the water is advected upstream and its mean salt content with it. This is the Stokes drift associated with a finite amplitude tidal-wave propagating back and forth in the channel. At station A, this term is higher during the spring tide survey than in the neap survey, 23.87±0.80 and 2.95±0.10 kgm^−1^s^−1^, respectively. At station B this term is almost zero, presenting a maximum and minimum value of 0.94±0.06 and 0.43±0.01 kgm^−1^s^−1^ during the August and January surveys, respectively. These values may be explained by the tidal wave characteristics in this region of the channel. Station B is located near the channel's head (close to the river's mouth) and as [5] found in their work, as the tidal wave propagates from deeper toward shallow areas of the lagoon, the tide is reflected from the shore and changes from a progressive to a standing wave. In the case of a standing wave, tidal elevation and tidal velocity has a a phase difference of 90°, and the tidally-averaged Stokes drift vanishes. In station B the Stokes drift is positive since at this point the tidal wave has a progressive and a standing component. This feature is induced not only by the location of the survey point, which is placed on a shallow area, but also by the presence of a dam 2 km upstream. Therefore, the Stokes wave transport presents positive values (close to zero), indicating an upstream tidal wave transport.

The dispersive tidal correlation term (c) switches sign from the colder to the warmer season surveys. During the January and December surveys, this term (c) is positive contributing to the upstream salt transport. The opposite, a downstream contribution to the salt transport, occurs during the June and August surveys. At station A, this value is maximum during the February 28^th^ survey, (14.42±0.82 kgm^−1^s^−1^). According to [3] in an idealized well-mixed estuary the phase difference between *V_t_* and *S_t_* would be 90°, since the maximum salinity is reached at the end of the flood tide, and therefore the contribution of this term to the net transport would be zero since their integral in quadrature is zero. [26] suggested that trapping of water by topographic irregularities could lead to a phase difference smaller than 90°. In the case of Espinheiro channel with a major channel and several side branches, the tidal elevation and velocities are not in phase in the main channel. The momentum of the flow in the main channel causes the current to continue to flow against an opposing pressure gradient. In contrast, the side channels have less momentum and the current direction changes when the water level begins to drop. At this stage, the water in the side channels recedes to the main stream in a different tidal instant, leading to a phase difference between *V_t_* and *S_t_* smaller than 90°. This can explain the positive sign of the colder season's surveys, but not the negative values during the summer surveys. If the phase difference is larger than 90°, term (c) will be negative, as observed by [1] and [3]. This feature can be observed in two-layered estuaries, where the longitudinal salinity gradients are smaller in the lower layer than in the upper layer of the estuary. Consequently, the tidal salinity oscillation is reduced in the lower layer and the tidal current near the bottom leads the tidal current in the upper layer, thus ebbing earlier in the bottom layer. The depth averaged velocity *V_t_* will lead *S_t_* by an average of the vertical phase profile. In contrast, due to its smaller bottom gradient, the depth averaged salinity will be little influenced by the bottom layer salinity oscillation and will follow the surface salinity values. This makes the phase difference larger than 90° with the consequent negative values for term (c).

Term (d) which represents the density driven circulation, is also positive, contributing to a landward (dispersive) salt transport. At station A, this term is residual, presenting values of 0.35±0.01 (February 21) and 0.04±0.01 kgm^−1^s^−1^) (February 28). These values confirm the analysis made in the currents and salinity section results where the non-tidal velocity and salinity figures reveal almost no density driven circulation. At station B, term (d) is one of the most important terms driving the salt transport. A pronounced density driven circulation is observed in all surveyed periods as depicted in Figures 4 and 5. This term is higher during the January and December surveys, reaching a maximum of 6.85±0.18 kgm^−1^s^−1^ during the December survey. The minimum value is found during the August survey with a value of 3.48±0.17 kgm^−1^s^−1^. At station B, this term increases by at least one order of magnitude when compared to the results at station A. At this location, when the freshwater discharge increases, the compensating bottom current also increases carrying salt up the estuary, thus increasing the cross-sectional salt transport.

Term (e), which is associated with tidal shear or unsteady wind effect, is not relevant for the transport of salt at station A. In fact, at this station, the results reveal marginally small values for terms (e), (f) and (g) when compared to the other terms. However, at station B, the results show that this term has the same order of magnitude as the gravitational circulation term for the colder seasons surveys (January and December), presenting values of 3.79±0.05 (January) and 1.51±0.02 kgm^−1^s^−1^ (December). This term is positive and dispersive for all the surveys. Terms (f) and (g) present residual values for all the periods surveyed. These terms are dependent on the tidal amplitude and their small values are due to the moderate tidal amplitude of the forcing tidal wave.

All the results reveal an imbalance in the net advective salt budget and during all the survey periods the channel was exporting salt. This imbalance may be due to a wrongly assumed lateral homogeneity of the locations surveyed. Another fact that can influence the results is the channel location in the very complex central area of the Ria de Aveiro lagoon, with the study area located within a section of the main channel with several side channels complicating the circulation patterns. The results may also be strongly dependent on the wind conditions, as local prevailing winds act down-gradient and tend to increase the density driven circulation, leading to positive dispersion values. As [3] refers in his work, the loss of salt by the channel is probably affected by large-scale wind gradients over all the lagoon and adjacent continental shelf which drive salt transport toward the ocean. Such wind-driven effect is not fully captured by the terms (a) and (b) and a much longer time series is needed to determine its behavior.

### Conclusions

This work analyses the importance of each of the terms of the salt transport equation as a proxy for the transport of dissolved substances. Negative salt transport indicates the seaward flushing rate of river-borne substances, and positive salt transport the upstream advection or upstream dispersion of oceanic water. A similar approach is made in [27] using the salt transport equation to calculate nitrogen fluxes in a mangrove area.

The two locations studied in this work are sensitive and important areas of the Espinheiro channel. Station A, located near the channel's mouth is important because there is only a single connection between the inner channel and the adjacent ocean and the channel's dynamics is strongly influenced by the tidal flow induced by the Atlantic tide. Station B is located near the channel's head in an ecologically sensitive area, close to Rio Vouga's mouth, where the existence of a paper mill constitutes an important environmental risk. Hence, the understanding of the mechanisms leading to the spreading and flushing of dissolved substances is relevant.

Near the mouth of the channel, the main contributions to the salt transport are from the freshwater discharge (terms (a)), Stokes drift (term (b)) and the tidal correlation terms (term (c) of equation 6). The net salt transport accounts for more than 80% of the downstream salt transport driven by the freshwater discharge. Near the channel’s head (at Station B), the main contributions to the salt transport are the freshwater discharge and the density driven circulation terms. At this location, on average, the net salt transport accounts for about 31% of the salt flushed by the river. Moreover, it was found that the residual circulation terms are major contributors to the salt transport.

The predominance in station A of the bulk input of freshwater volume (terms a and b of equation 6) as the main mechanism for the spreading of river-borne substances and the flushing of the lagoon, indicate that after an hazardous discharge of contaminants, the river flow should be kept as high as possible to allow a swift flushing of contaminants. A temporary isolation of the side channels will reduce the topographic trapping mechanism (term c in equation 6) and contribute to the flushing of the lagoon.

In station B, increasing river discharge will also help flush the lagoon of river-born contaminants. However, due to the importance of the density-driven circulation, there will be a net upstream import of oceanic water and any contaminants dissolved therein.

In dry months and in systems similar to the Ria de Aveiro, upstream industry and agriculture store freshwater behind a system of dams to prevent salt intrusion. This practice will lead to an artificial downstream increase of salinity, thus stressing the ecosystem. In these cases, an environmental minimum freshwater flow is recommended. According to our results, the timing of the release of this minimum flow is important to the persistence of the desired effect. Hence, in the upstream reaches of the lagoon, the release of freshwater under spring tides and high winds will reduce the density-driven circulation term, prolonging the persistence of freshwater inside the system.

In the absence of sampling errors and assuming a steady condition, the sum of all terms of the salt transport equation would be zero. However this study found a salt imbalance during all surveys meaning that the estuary is in fact exporting salt. The rates are presented in Table 3, indicating a seaward salt transport.

Although these short term surveys (only one or two tidal cycles) fail to give information about meteorological effects on salt transport, they can produce valuable results about processes on tidal and subtidal time scales. Sampling strategies like the one followed in the data acquisition, with just one station in the middle of the channel, without assessing the results against the tidal strength (neap and spring periods), changes in the bathymetry or magnitude of the longitudinal density gradient, could lead to a salt transport value which results in a number only meaningful for the specific conditions under which the measurements where made [28]. However, our analysis and contextualization of the several terms of the transport equation in two very different areas of the estuarine system helps to identify the vulnerability of the system to risks of river-borne (e.g. chemical contaminants from the nearby industry) and oceanic pollutants and how strategies may change according to the area at risk.
